# Evaluation of epidermal growth factor receptor signaling effects in gastric cancer cell lines by detailed motility-focused phenotypic characterization linked with molecular analysis

**DOI:** 10.1186/s12885-017-3822-3

**Published:** 2017-12-13

**Authors:** Simone Keller, Julia Kneissl, Verena Grabher-Meier, Stefan Heindl, Jan Hasenauer, Dieter Maier, Julian Mattes, Peter Winter, Birgit Luber

**Affiliations:** 1 Institut für Allgemeine Pathologie und Pathologische Anatomie, Technische Universität München, Klinikum rechts der Isar, Trogerstr. 18, 81675 München, Germany; 2 Institute of Computational Biology, Helmholtz Zentrum München-German Research Center for Environmental Health, Ingolstädter Landstr. 1, 85764 Neuherberg, Germany; 3 Technische Universität München, Center for Mathematics, Chair of Mathematical Modelling of Biological Systems, Boltzmannstraße 3, 85748 Garching, Germany; 4 Biomax Informatics AG, Robert-Koch-Str. 2, 82152 Planegg, Germany; 5 Knowledge-Based Vision Systems, Software Competence Center Hagenberg GmbH, Softwarepark 21, 4232 Hagenberg, Austria; 6 GenXPro GmbH, Altenhöferallee 3, 60438 Frankfurt am Main, Germany; 7 Present Address: MATTES Medical Imaging GmbH, Softwarepark 21, 4232 Hagenberg, Austria

**Keywords:** EGFR, Cetuximab, Motility, Invasion, Gastric cancer

## Abstract

**Background:**

Gastric cancers frequently overexpress the epidermal growth factor receptor (EGFR), which has been implicated in pathological processes including tumor cell motility, invasion and metastasis. Targeting EGFR with the inhibitory antibody cetuximab may affect the motile and invasive behavior of tumor cells. Here, we evaluated the effects of EGFR signaling in gastric cancer cell lines to link the phenotypic behavior of the cells with their molecular characteristics.

**Methods:**

Phenotypic effects were analyzed in four gastric cancer cell lines (AGS, Hs746T, LMSU and MKN1) by time-lapse microscopy and transwell invasion assay. Effects on EGFR signaling were detected using Western blot and proteome profiler analyses. A network was constructed linking EGFR signaling to the regulation of cellular motility.

**Results:**

The analysis of the effects of treatment with epidermal growth factor (EGF) and cetuximab revealed that only one cell line (MKN1) was sensitive to cetuximab treatment in all phenotypic assays, whereas the other cell lines were either not responsive (Hs746T, LMSU) or sensitive only in certain tests (AGS). Cetuximab inhibited EGFR, MAPK and AKT activity and associated components of the EGFR signaling pathway to different degrees in cetuximab-sensitive MKN1 cells. In contrast, no such changes were observed in Hs746T cells. Thus, the different phenotypic behaviors of the cells were linked to their molecular response to treatment. Genetic alterations had different associations with response to treatment: while *PIK3CA* mutations and *KRAS* mutation or amplification were not obstructive, the *MET* mutation was associated with non-response.

**Conclusion:**

These results identify components of the EGFR signaling network as important regulators of the phenotypic and molecular response to cetuximab treatment.

**Electronic supplementary material:**

The online version of this article (10.1186/s12885-017-3822-3) contains supplementary material, which is available to authorized users.

## Background

Gastric cancer is the fifth most common malignancy and the third leading cause of cancer death worldwide [1]. Progress in the treatment of gastric cancer has been limited due to molecular and clinical heterogeneity [2]. As a consequence of the molecular diversity of gastric cancer, personalized therapy with targeted agents is an important step to improve the outcome of patients with this common disease [2]. Receptor tyrosine kinase (RTK)/RAS alterations occur frequently in gastric cancer and lead to the definition of five distinct gastric cancer subgroups characterized by genomic alterations in *FGFR2*, *KRAS*, *EGFR*, *ERBB2/HER2* and *MET*, suggesting that approximately 37% of patients may potentially be treatable by RTK/RAS-directed therapies [3]. The targeted agents that are currently approved for gastric cancer treatment include the HER2-targeted monoclonal antibody, trastuzumab, and the vascular endothelial growth factor receptor 2 (VEGFR2)-targeted monoclonal antibody, ramucirumab. EGFR, ERBB3/HER3, VEGF, PI3K/mTOR, FGFR2 and MET show promise as new targets for gastric cancer treatment [2].

The frequent overexpression of EGFR in gastric cancer generated particular interest in this RTK for targeted therapy. However, in the clinical EXPAND trial, the EGFR-inhibitory monoclonal antibody cetuximab, in combination with capecitabine and cisplatin, showed no survival benefits compared to chemotherapy alone in patients with advanced or metastatic gastric or esophagogastric cancer [4]. Because of the heterogeneity of gastric cancer, further molecular classification of the disease may be required [4].

Genetic alterations in the EGFR signaling network and in the direct or indirect interaction partners of EGFR (e.g., RTKs or cell adhesion molecules) were shown to be involved in the response to EGFR-targeted therapies [5, 6]. We previously reported that high expression levels of EGFR and low levels of constitutive EGFR activation were positively correlated with cetuximab sensitivity, which was determined via cell proliferation assays, whereas the activation of the RTK, MET, and mutations in *KRAS* or *CDH1* were associated with cetuximab non-responsiveness using gastric cancer cell lines as a model system [6, 7]. During malignant growth, cells must acquire the capability to sustain proliferative signaling, evade growth suppressors, resist cell death, enable replicative immortality, induce angiogenesis and activate invasion and metastasis [8]. Therefore, our previous studies were limited by the fact that we focused on only cell proliferation as a readout of successful cetuximab treatment.

EGFR signaling is the growth factor system most often implicated in tumor progression through the activation of the receptor or its ligands, which leads to both mitogenesis and motility that correlate with tumor progression [9–12]. EGFR overexpression results in increased tumor cell motility in vivo and is associated with enhanced intravasation and metastasis [13].

The aim of our study was to analyze the effects of EGFR signaling in a panel of four human EGFR-expressing gastric cancer cell lines (AGS, Hs746T, LMSU and MKN1) by detailed characterization of the link between the differing motility-focused phenotypic behaviors of the individual cell lines and their specific molecular characteristics. In a recent study using a cell proliferation assay, we demonstrated that MKN1 cells were sensitive to cetuximab under single-agent treatment conditions, whereas AGS, Hs746T and LMSU cells were insensitive [7]. Here, we assessed the effect of treatments with EGF, cetuximab or combinations of both in the four cell lines using additional phenotypic assays (motility assay and invasion assay) and compared these results with the results obtained from the proliferation assay. Furthermore, we analyzed the activation of key EGFR signaling pathway molecules in a single cetuximab-responsive (MKN1) and cetuximab-resistant (Hs746T) cell line.

## Methods

### Cell lines and cultivation conditions

The human gastric cancer cell lines AGS, Hs746T, LMSU and MKN1 were used. As reported previously, AGS cells were obtained from the European Collection of Cell Cultures (ECACC, catalogue number 89090402), a Health Protection Agency Culture Collection supplier of authenticated and quality-controlled cell lines and nucleic acids (Porton Down, Salisbury, UK; http://www.hpacultures.org.uk/collections/ecacc.jsp). MKN1 (catalogue number RCB1003) and LMSU (catalogue number RCB1062) cells were supplied by the cell bank, RIKEN BioResource Center (Tsukuba, Japan). Hs746T cells were obtained from the ATCC Cell Biology Collection (LGC Standards GmbH, Wesel, Germany, catalogue number ATCC HTB-135) [6, 7].

AGS and MKN1 cells were grown in RPMI 1640 medium (Life Technologies, Darmstadt, Germany) supplemented with 2 mM L-glutamine (Life Technologies) as previously reported [6]. Hs746T cells were cultured in Dulbecco’s Modified Eagle Medium (DMEM) with GlutaMAX™-I, 4500 mg/l D-glucose and sodium pyruvate (Life Technologies) and LMSU cells in Nutrient Mixture F-10 Ham medium (Sigma-Aldrich) as previously described [7]. All cell culture media were supplemented with 10% fetal bovine serum (FBS) *Sera Plus* (PAN-Biotech, Aidenbach, Germany) and with penicillin-streptomycin (PAA Laboratories, Pasching, Austria; 100 IU/ml, 100 μg/ml). After thawing frozen cells, the absence of mycoplasma in the conditioned medium was routinely confirmed.

### Time-lapse microscopy

For live-cell imaging, 35-mm glass bottom culture dishes (MatTek Corporation, Ashland, MA, USA) were coated with either 100 μg/ml collagen type I (BD Biosciences, Heidelberg, Germany) for 30 min at 37 °C or with 10 μg/ml fibronectin (Sigma-Aldrich, Steinheim, Germany) for 90 min at room temperature. AGS, Hs746T and MKN1 cells were seeded onto collagen I-coated plates and LMSU cells on fibronectin-coated plates, according to the ability of the cell lines to adhere and move on different matrices. Cells were seeded at densities of 1.7–3.0 × 10^5^ cells/plate, depending on the cell line. The medium was changed 1 h after seeding, to eliminate non-adhesive cells. Next, medium containing FCS was added and cells were stimulated with 5 ng/ml EGF (Sigma-Aldrich) and/or cetuximab (concentrations: 0.05, 0.1, 1, and 50 μg/ml; Merck, Darmstadt, Germany). Further cultivation was achieved in a microscope-coupled incubation chamber (5% CO_2_, 37 °C). Time-lapse video observations began 2 h after cell seeding. Phase-contrast images were taken every 3 min for 7 h with an Axiovert laser scanning microscope LSM 510 (Zeiss, Jena, Germany) with a PNF 20×/0.4 PH2 objective lens and a helium-neon laser at 543 nm in transmission scanning mode or the Axio Observer A1 microscope (Zeiss) with a 10×/0.3 Ph1 objective lens. As previously reported [14], the “percentage of motile cells” and the “average cell speed” were analyzed.

### Matrigel invasion assay

The two-chamber transwell system (BD Biosciences) for invasion assays was rehydrated for 2 h in medium without FBS at 37 °C, 5% CO_2_. Approximately 1 × 10^4^ cells were seeded into 500 μl medium without FBS, and cells were incubated for 4 h. Subsequently, cells were treated with combinations of 5 ng/ml EGF and/or cetuximab (concentrations: 0.1, 1 and 50 μg/ml cetuximab). As a chemoattractant, 0.1% FBS was added to the lower chamber. The cells were incubated for an additional 22 h. The assay was performed according to the manufacturer’s instructions. The “relative invasiveness” was determined by counting all cells fixed at the transwell membrane under the microscope.

### Western blot analysis

Western blot analyses were performed as previously reported [6, 7]. The cells were stimulated for 1, 3, or 15 min or 4 h with 5 or 30 ng/ml EGF and/or 0.05, 0.1, 1, or 10 μg/ml cetuximab. A total of 30 μg of total proteins were loaded per well. Antibodies were used under the following conditions: anti-pEGFR (Life Technologies, Darmstadt, Germany; # 44788G; dilution 1:2000 in 5% milk/TBS-T), anti-pMAPK (Cell Signaling Technology (CST), distributed by New England Biolabs in Frankfurt, Germany; # 9101; dilution 1:2000 in 5% milk/TBS-T), anti-pAKT (CST; # 9271; dilution 1:2000 in 5% BSA/TBS-T), anti-α-tubulin (Sigma-Aldrich; # T9026; dilution 1:10,000 in 5% milk/TBS-T), anti-pCREB (CST; # 4276; dilution 1:1000 in 5% milk/TBS-T), anti-pFAK (BD Biosciences; # 611723; dilution 1:1000 in 5% BSA/TBS-T), anti-mouse (GE Healthcare, distributed by VWR in Ismaning, Germany; # NA931; dilution 1:10,000 in 5% milk/TBS-T) and anti-rabbit (CST; # 7074; dilution 1:2000 in TBS-T). For signal quantification, signals were analyzed by densitometry using ImageJ 1.44p Software (National Institutes of Health, Bethesda, Maryland, USA). Concerning the results shown in Figs. 4 and 5, each membrane was probed with antibodies specific for pEGFR (Y1068), pAKT (S473) and α-tubulin (loading control). Each blot was then stripped and re-probed with an antibody specific for pMAPK (T202/Y204). One representative Western blot analysis for each stimulation period is shown.

### Proteome profiler

The Human Phospho-Kinase Array Kit (R&D Systems, Wiesbaden-Nordenstadt, Germany) was used to perform the proteome profiler analysis. For the preparation of total protein extracts, 3 × 10^6^ cells were seeded into medium containing 10% FBS. After 6 h incubation, cells were washed twice with 1× PBS, and medium without FBS was added. The cultures were incubated overnight. Afterwards, cells were treated with 30 ng/ml EGF and/or 1 μg/ml cetuximab for 4 h. The preparation of cellular extracts and the proteome profiling was carried out according to the manufacturer’s instructions, with a total protein amount of 250 μg per assay. For data evaluation, signals were analyzed by densitometry with ImageJ and were normalized to the assay-internal negative and positive controls as well as the overall signal of each phosphorylation site (= 100%).

### Statistical analysis

Correlation analysis between the parameters “percentage of motile cells” and “average speed” was performed using Pearson correlation coefficient and Spearman’s rho test. A two-sided *p*-value ≤0.05 was considered to be statistically significant. Pairwise comparisons between different treatment conditions were performed using the two-sided Welch t-test. One sample t-test was used to test the activity ratio of treated samples to untreated samples against a reference value of 100%, which indicated equality of activity. Data analyses were performed on an explorative significance level of 0.05 using the statistical software R (The R Foundation for Statistical Computing, Vienna, Austria) and IBM SPSS Statistics 22 and 23 (IBM, Armonk, NY, USA). Statistical values at a significance level ≤ 0.05 are indicated by * and ≤0.01 by **. A summary of all statistical data is available from the authors upon request.

### Construction of a network connecting EGFR signaling to the regulation of cellular motility

To detect potential directly protein-based and therefore fast-acting mechanisms involved in EGFR-dependent changes in motility, beyond the commonly implicated slow-acting transcriptional effects, we integrated data from the KEGG and Reactome pathways and BioModels SBML models for EGFR signaling that describe the regulation of cellular motility, regulation of actin and myosin and regulation of intercellular adhesion. In addition, we curated several components from the literature (e.g., MET, FAK) implicated in EGFR signaling or cell motility but not part of the corresponding standard pathways or models.

To connect these components with other nodes, we extended the network to include human protein-protein interaction and transcriptional regulation data integrated from DIP, IntAct and MINT for PPI and from ITFP [15] for transcriptional regulation, respectively.

### Database search for genetic alterations

Mutational data were derived from the last public COSMIC [16] version (v71), the 439 TCGA (http://cancergenome.nih.gov/, The Cancer Genome Atlas Home Page. In: The Cancer Genome Atlas - National Cancer Institute [Internet]) stomach adenocarcinoma samples available in July 2015 and canSAR in October 2014.

## Results

We initially conducted phenotypic analysis to determine the cetuximab sensitivity of the gastric cancer cell lines. All analyzed cell lines expressed the EGFR at different levels as previously shown [7].

### Basal levels of gastric cancer cell motility

First, the basal levels of gastric cancer cell motility were determined using time-lapse microscopy and quantification of the two-dimensional (2D) migration behavior based on two parameter values (“percentage of motile cells”, “average speed”) for each cell.

**Fig. 1:**
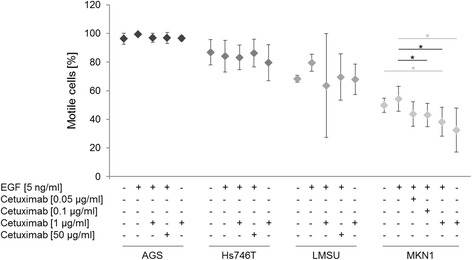
Effect of EGF and/or cetuximab treatment on the percentage of motile cells. To determine the percentage of motile cells, between 146 and 291 cells obtained from three to eight movies were analyzed per cell line and condition by time-lapse microscopy. Cells were grown in medium containing FBS. *P*-values at significance levels of ≤0.050 are indicated by (*). Comparisons that are both significant and relevant for the definition of responder and non-responder cell lines are marked with black bars and asterisks, whereas comparisons that are only significant are marked with gray bars and asterisks. Details on the number of analyzed movies and the mean and standard deviation (SD) of the percentage of motile cells are presented in Additional file 1: Table S1, and *p*-values are listed in Additional file 1: Table S2

**Fig. 2:**
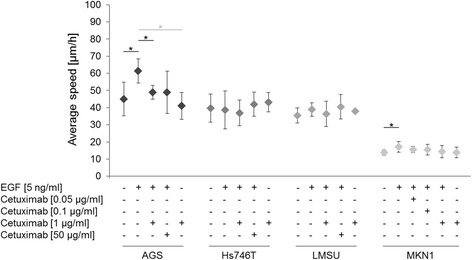
Effect of EGF and/or cetuximab treatment on the average speed [μm/h]. For the determination of the descriptor “average speed”, cell numbers and conditions were identical to those in Fig. 1. P-values at significance levels of ≤0.050 are indicated by (*). Comparisons that are both significant and relevant for the definition of responder and non-responder cell lines are marked with black bars and asterisks, whereas comparisons that are only significant are marked with gray bars and asterisks. Details on the numbers of movies and the mean and SD of the average speed are shown in Additional file 1: Table S1, and p-values are listed in Additional file 1: Table S3

Striking differences in the percentage of motile cells were observed between the various cell lines without treatment (Fig. 1, Additional file 1: Table S1): 96.26% (AGS), 88.84% (Hs746T), 68.29% (LMSU) and 49.81% (MKN1). These differences in the proportion of motile cells were reflected by a wide range of the mean average speed of untreated cells (Fig. 2, Additional file 1: Table S1): 45.00 μm/h (AGS), 39.73 μm/h (Hs746T), 35.44 μm/h (LMSU) and 13.88 μm/h (MKN1). The correlation of the percentage of motile cells with average speed was strong (*n* = 4; Pearson test *p* = 0.052, correlation = 0.948; Spearman’s rho test *p* = 0.01, correlation coefficient = 1.000).

To summarize, under the chosen experimental conditions the gastric cancer cell lines AGS, Hs746T, LMSU and MKN1 differed in their 2D motility as shown by two different but correlated descriptors, the percentage of motile cells and the average speed.

### Motile behavior of gastric cancer cell lines in response to EGF and/or cetuximab

One of the biological responses to EGFR signaling is enhanced cellular motility [17]. The effect of EGF and/or cetuximab on the motile behavior of the cells was determined using time-lapse microscopy as described above. Cells were treated with EGF at a concentration of 5 ng/ml and varying concentrations of cetuximab (0.05–50 μg/ml). We analyzed 1) whether the four cell lines reacted to EGF treatment with enhanced cellular motility and 2) whether this EGF-enhanced cellular motility was inhibited by cetuximab at a statistically significant level. If these two conditions were met, we considered a cell line “responsive” or “sensitive” to cetuximab treatment with regard to the motile behavior. Again, the percentage of motile cells and average speed were used as surrogate markers for cellular motility.

Comparisons of untreated and EGF-treated cells as well as EGF-treated and EGF/cetuximab-treated cells revealed no significant changes in the percentage of motile cells in the cell lines AGS, Hs746T and LMSU (Fig. 1). In MKN1 cells, EGF treatment increased the percentage of motile cells. Treatment with additional cetuximab led to a significant concentration-dependent decrease (0.1 and 1 μg/ml cetuximab (*p* = 0.036, *p* = 0.013, respectively), Additional file 1: Table S2). In line with the definition above, MKN1 cells were considered cetuximab-responsive for the parameter percentage of motile cells. The effect of cetuximab-alone treatment on the proportion of motile cells, as an additional control for treatment susceptibility of the basal level of cellular motility, was not statistically significant for any of the analyzed cell lines.

Two cell lines reacted to EGF treatment with increased average speed compared to untreated cells: AGS (*p* = 0.023) and MKN1 cells (*p* = 0.038) (Fig. 2, Additional file 1: Table S3). Significant reversion of the EGF-induced increase in average speed was observed in the cell line AGS at 1 μg/ml cetuximab (*p* = 0.030) compared to EGF-treated cells. In MKN1 cells, a concentration-dependent decrease of the average speed due to cetuximab treatment was observed but was not statistically significant. In consequence, AGS cells were considered as a motility responder cell line. No remarkable changes in average speed were observed in Hs746T and LMSU cells upon EGF and/or cetuximab treatment. No effects of cetuximab-alone treatment on average speed were observed in the cell lines when compared with untreated cells.

Based on these results, and following the definition of response or sensitivity described above, the cell lines AGS and MKN1 were classified as cetuximab-responsive with regard to their average speed (AGS) or percentage of motile cells (MKN1), respectively, whereas Hs746T and LMSU cells were not found to be sensitive to cetuximab treatment.

### Morphological effects of EGF and/or cetuximab treatment in MKN1 and Hs746T cells

Treatment of cells with EGF induced receptor dimerization and the activation of an integrated network that mediated the reorganization of the actin cytoskeleton [18]. This resulted in rapidly detectable alterations of the morphology of cells, including membrane ruffles and lamellipodia [18, 19]. Morphological changes after EGF and/or cetuximab treatment were compared in the cetuximab-responsive cell line MKN1 and in non-responsive Hs746T cells during a 7 h time-lapse movie.

Morphologically, MKN1 cells showed enhanced formation of lamellipodia and a slight increase in filopodia development upon EGF treatment compared to the untreated control (Additional file 2: Figure S1). Both EGF-induced effects were inhibited by concomitant application of cetuximab at a concentration of 1 μg/ml. Cetuximab-alone treatment resulted in fewer membrane protrusions compared to untreated cells. No difference between the several treatments was observed for Hs746T cells.

### Invasive behavior of gastric cancer cell lines in response to EGF and/or cetuximab

Cell invasion is another crucial step for the formation of metastases [20]. The ability of cells to actively invade the surrounding tissue strongly depends on the proteolytic digestion of the extracellular matrix components. In addition to time-lapse microscopy, we performed transwell cell invasion assays.

**Fig. 3:**
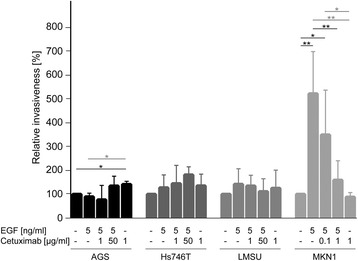
Effect of EGF and/or cetuximab treatment on the relative invasiveness. For the determination of the relative invasiveness of the gastric cancer cells, two-chamber assays were used. Cells were treated as indicated for 22 h. FBS (0.1%) was used as a chemoattractant. The mean value of three independent experiments is shown. *P*-values at significance levels of ≤0.050 and ≤0.010 are indicated by (*) and (**), respectively. Comparisons that are both significant and relevant for the definition of responder and non-responder cell lines are marked with black bars and asterisks, whereas comparisons that are only significant are marked with gray bars and asterisks. P-values are listed in Additional file 1: Table S4

As shown in Fig. 3 and Additional file 1: Table S4, a strong increase in the number of invasive MKN1 cells was detected after EGF stimulation (*p* = 0.006). This increase was reduced by concomitant application of cetuximab in a concentration-dependent manner that almost completely reverted by the application of 1 μg/ml cetuximab to EGF-treated MKN1 cells (*p* = 0.007). No effect of exclusive cetuximab treatment compared to the untreated control was observed. AGS, Hs746T and LMSU cells did not show any remarkable changes in invasion after treatment with either EGF or cetuximab.

Together, a significant EGF-induced increase in the number of invasive MKN1 cells was detected that was reverted by concomitant cetuximab treatment in a concentration-dependent manner (Table 1). In contrast, AGS, Hs746T and LMSU cell lines did not react as responders in the invasion assay.

**Table 1:** Summary of the phenotypic evaluation of cetuximab sensitivity of gastric cancer cell lines using different assays

Cell line	Proliferation^a^	Motility^b^		Invasion^c^
	Viability^d^	Motile cells (%)^e^	Average speed^e^	Invasive cells^e^
AGS	–	–	+	–
Hs746T	–	–	–	–
LMSU	–	–	–	–
**MKN1**	**++**	**+**	**–**	**+**

^a^determined via XTT cell proliferation assay as previously published by Kneissl et al. [7]

^b^determined via time-lapse microscopy

^c^determined via Matrigel invasion assay

^d^- = not detectable; +/++/+++ = detectable at low/intermediate/high levels as previously published by Kneissl et al. [7]

^e^ + = EGF-induced increases and significant reversions by EGF/cetuximab treatment at a significance level of at least ≤0.050

### Time-resolved activation profile of EGFR and its downstream effectors MAPK and AKT

Based on current knowledge, EGFR or HER2 is the main driver of downstream signaling in cancers that are highly sensitive to EGFR or HER2 inhibitors, primarily via the MAPK and the phosphatidylinositol 3-kinase (PI3K)/AKT pathways [21, 22]. In consequence, inhibition of EGFR in our gastric cancer model should inhibit these two downstream pathways in a cetuximab-sensitive cell line but not in a non-responder line. Furthermore, the MAPK and the PI3K/AKT pathways have been implicated in the regulation of cellular motility [23, 24]. To link the phenotypic data obtained in this study with molecular changes, we determined the effect of EGF and/or cetuximab treatment on the activation status of EGFR, MAPK and AKT.

We previously examined the effects of EGF and cetuximab treatment for 3 min on the expression and activation level of EGFR in gastric cancer cell lines [7].

In the present study, the time-resolved (1 min, 3 min, 15 min, and 4 h) activation profiles of EGFR and its downstream effectors MAPK and AKT were compared in the responder cell line MKN1 and the non-responder cell line Hs746T under different treatment conditions (EGF and/or cetuximab) by Western blot analysis.

**Fig. 4:**
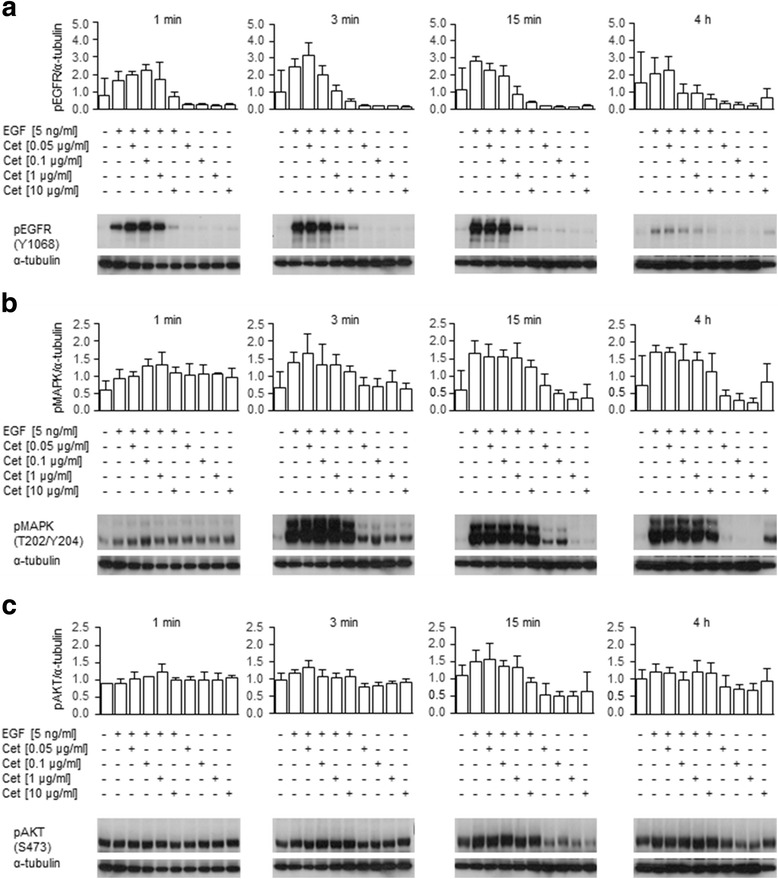
Effect of EGF and/or cetuximab treatment on EGFR, MAPK and AKT activation in MKN1 cells. EGFR, MAPK and AKT activation were detected in total lysates of EGF- and/or cetuximab-treated cells by Western blot analysis using phospho-specific antibodies. Each membrane was probed with antibodies specific for **a** pEGFR (Y1068), **c** pAKT (S473) and α-tubulin (loading control). Each blot was then stripped and re-probed with an antibody specific for **b** pMAPK (T202/Y204). One representative Western blot analysis for each stimulation period is shown. The depicted results are representative of three independent experiments. The average phosphorylation levels were quantified using densitometric analysis and calculated in relation to the levels of α-tubulin (+SD). Statistical *p*-values are listed in Additional file 1: Table S5. Additionally, one representative Western blot of each stimulation period is shown. Abbreviation: Cet = cetuximab

In MKN1 cells, the EGF-induced activation of EGFR that was inhibited by cetuximab in a concentration-dependent manner was detected at all stimulation times (Fig. 4a). Levels of phosphorylated EGFR were significantly lower under treatment with 1 or 10 μg/ml cetuximab at two time points (3 min and 15 min) compared to EGF-alone treatment (Additional file 1: Table S5). Upon EGF treatment, MAPK activation (Fig. 4b) and minor AKT activation (Fig. 4c) were induced, which were not fully reversed by additional cetuximab treatment under the chosen experimental conditions. The fact that AKT was already active without EGF stimulation may be because MKN1 cells contained an activating *PIK3CA* mutation [16]. Effects of cetuximab-alone treatment on the level of phosphorylated EGFR and phosphorylated MAPK were observed but were not statistically significant. Single cetuximab treatment for either 15 min or 4 h reduced AKT activation in MKN1 cells. The statistical values are presented in Additional file 1: Table S5.

**Fig. 5:**
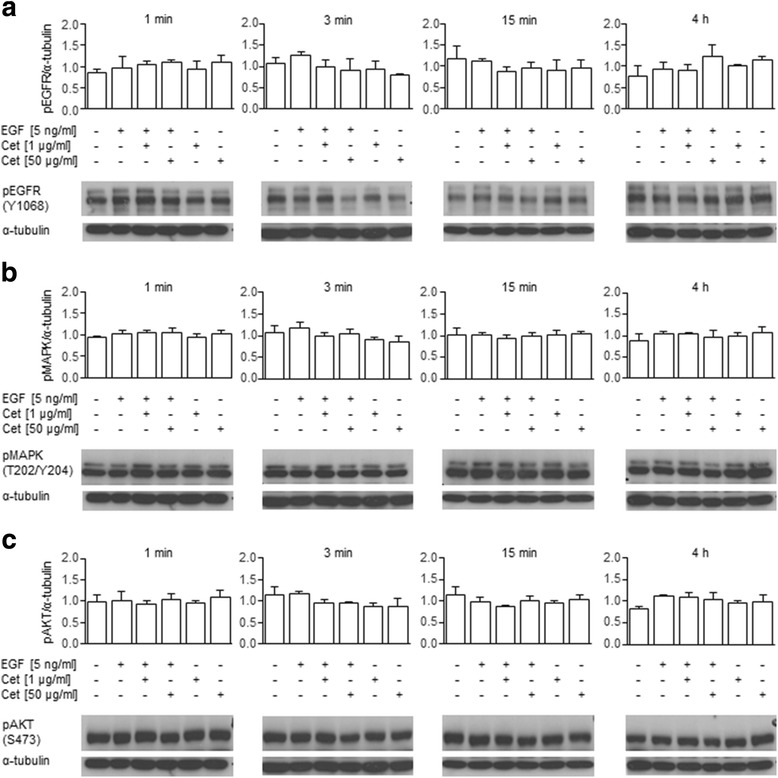
Effect of EGF and/or cetuximab treatment on EGFR, MAPK and AKT activation in Hs746T cells. Phosphorylation of EGFR was detected in total cell lysates by Western blot analysis using antibodies directed against **a** pEGFR (Y1068), **b** pMAPK (T202/Y204) and **c** pAKT (S473). α-tubulin was used as a loading control. The depicted results are representative of three independent experiments. Each membrane was probed with antibodies specific for **a** pEGFR (Y1068), **c** pAKT (S473) and α-tubulin (loading control). Each blot was then stripped and re-probed with an antibody specific for **b** pMAPK (T202/Y204). One representative Western blot analysis for each stimulation period is shown. The average phosphorylation levels were quantified using densitometric analysis and were calculated in relation to the levels of α-tubulin (+SD). Statistical p-values are listed in Additional file 1: Table S6. Additionally, one representative Western blot of each stimulation period is shown. Abbreviation: Cet = cetuximab

In Hs746T cells (Fig. 5), all pathways were active in the absence of EGF, which indicated a possible constitutive activation of the EGFR signaling. In these cells, the activation of EGFR, MAPK or AKT, independent of the treatment periods, was not significantly impaired by cetuximab (Fig. 5, Additional file 1: Table S6).

Thus, EGF treatment caused transient activation of EGFR and MAPK in MKN1 cells that was reverted by concomitant cetuximab treatment to some extent but had only marginal effects on AKT signaling. In Hs746T cells, little to no effect on EGFR, MAPK and AKT activation was detected.

### Analysis of the effects of the EGF and/or cetuximab treatment on EGFR downstream signaling by proteome profiler analysis

**Fig. 6:**
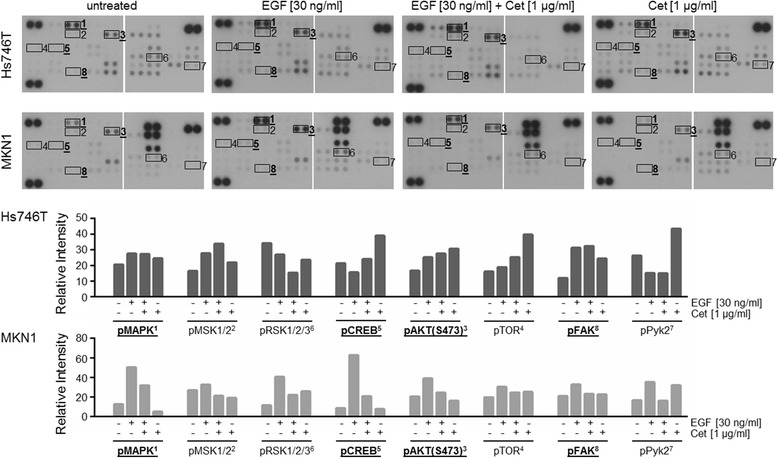
Phosphorylation profile of components of the EGFR signaling pathway in Hs746T and MKN1 cells. Levels of pMAPK (T202/Y204, T185/Y187), pMSK1/2 (S376/S360), pRSK 1/2/3 (S380), pCREB (S133), pAKT (S473), pTOR (S2448), pFAK (Y397) and pPyk2 (Y402) were determined via proteome profiler analysis. Cells were treated for 4 h with EGF and/or cetuximab. The average phosphorylation levels were quantified using densitometric analysis. The results for bolded and underlined phospho-proteins were confirmed via Western blot analysis (see Fig. 7)

To determine the effects of the EGF and/or cetuximab treatment on EGFR downstream signaling in MKN1 and Hs746T cells, a proteome profiler analysis analyzing the EGFR downstream kinase network was carried out (Fig. 6 and Additional file 3: Figure S2). These experiments focused on the late EGFR response to link the phenotype with the molecular results. Therefore, starved cells were treated for 4 h with EGF and/or cetuximab. We defined proteins as having an “EGF/cetuximab-responsive profile” if there was an increase in phospho-protein expression after EGF treatment that could be inhibited by concomitant cetuximab treatment.

MKN1 cells clearly showed an EGF/cetuximab-responsive profile for several components of both the MAPK signaling branch (pMAPK, pMSK1/2, pRSK1/2/3, and pCREB) and the AKT signaling pathway (pAKT and pTOR). In contrast, no such trend was detected for Hs746T cells.

FAK and Pyk2 are key regulators of cell migration [25]. Therefore, their phosphorylation profiles were of special interest. An EGF/cetuximab-responsive profile was detected for pFAK and pPyk2 in MKN1 but not in Hs746T cells.

Together, MKN1 cells clearly showed an EGF/cetuximab-responsive profile for several components of the MAPK and AKT signaling pathways in the proteome profiler analysis. No such result was obtained for Hs746T cells.

**Fig. 7:**
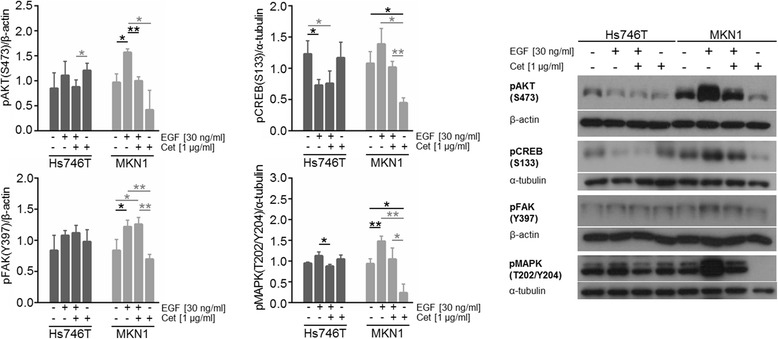
Verification of the results obtained by proteome profiler analysis. Levels of pAKT, pCREB, pFAK and pMAPK were determined via Western blot analysis. Equal loading of the lanes was confirmed by the detection of α-tubulin for pCREB and pMAPK and β-actin for pFAK and pAKT. The depicted results are representative of three independent experiments. P-values at significance levels of ≤0.050 and ≤0.010 are indicated by (*) and (**), respectively. Comparisons that are both significant and relevant for the definition of responder and non-responder cell lines are marked with black bars and asterisks, whereas comparisons that are only significant are marked with gray bars and asterisks. The average phosphorylation levels were quantified using densitometric analysis and calculated in relation to the levels of the loading control (+SD). Statistical p-values are listed in Additional file 1: Table S7. Additionally, one representative Western blot of each experiment is shown

To verify the proteome profiler findings, Western blot analysis for selected phospho-proteins (pAKT, pCREB, pFAK, pMAPK; Fig. 7) was performed. The experiments confirmed the findings of the proteome profiler for pAKT, pCREB and pMAPK in MKN1 cells and for pFAK for the most part (Additional file 1: Table S7). In accordance with the proteome profiler analysis, an EGF/cetuximab-responsive profile was not observed in Hs746T cells.

### Construction of a network connecting EGFR signaling to the regulation of cellular motility

Because the EGFR signaling network and direct or indirect interaction partners of EGFR are involved in the response to EGFR-targeted therapies [5–7], we aimed to determine how EGFR signaling is molecularly connected to the regulation of cell motility. To this end, a network was constructed from current knowledge about proteins implicated in either EGFR signaling or cytoskeleton regulation.

As shown in Additional file 4: Figure S3, 179 nodes from KEGG [26], Reactome [27], BioModels [28] and the literature are implied in EGFR signaling or cellular motility regulation events. To enrich the data analysis about potential regulatory interactions between EGFR signaling and the cytoskeleton, additional data about interactions between these nodes were derived from the protein-protein interaction databases MINT [29], DIP [30] and IntAct [31].

### Involvement of genetic alterations in the response to cetuximab

Data about somatic mutations and copy number changes were available from the integrated database canSAR for the AGS and MKN1 cell lines [32]. We extracted the most relevant data related to EGFR signaling, cetuximab sensitivity and the phenotypic behavior of the cells.

Our analysis of the sequencing results in the databases yielded the following mutations of genes contained in the previously derived network: *KRAS* was amplified (copy number 13, chromosome mean 3.19) and the gene copy numbers for *HRAS* and *NRAS* were elevated (copy number 3, chromosome mean 3.01 for both) in MKN1 cells. In addition, a mutation in phosphatidylinositol-4,5-bisphosphate 3-kinase, catalytic subunit alpha (*PIK3CA*) was described (p.E545K).

AGS cells contained several mutations in genes involved in the regulation of cellular motility, which might explain the result that 96% of cells were found to be motile: two mutations in the gene encoding fibroblast growth factor receptor 4 (*FGFR4*) (p.D425N and p.D465N) and single mutations in the genes *RHOA* (p.E64delE), *ACTN4* (p.S816C) and *CDH1* (p.G579 fs*9). A mutation in *KRAS* (p.G12D) was previously considered to cause cetuximab insensitivity of cells in a proliferation assay [6]. Here, mutations in *PIK3CA* were also reported (p.E453K, p.E545A).

Neither of the cell lines contained mutations or amplifications of the EGFR family members *HER2*, *HER3* or *HER4*. In contrast, the gene copy number of *EGFR* in both cell lines is elevated slightly (AGS cells: copy number 2, chromosome mean 2) or moderately (MKN1 cells: copy number 4, chromosome mean 3.78). AGS and MKN1 cells also did not contain mutations or amplifications of *MET*.

Hs746T cells have a constitutive MET activation [7], which is presumably caused by MET amplification [33]. Further statements as to the genetic characteristics cannot be made, except that *APC*, *MAP3K15* and *TP53* are also mutated. For LMSU cells, there was no available public data on somatic mutations and gene copy number alterations.

## Discussion

The evaluation of EGFR signaling effects in four gastric cancer cell lines (AGS, Hs746T, LMSU and MKN1) revealed that the MKN1 cell line (responder) was sensitive to EGF and/or cetuximab treatment in all phenotypic assays (proliferation assay [6, 7], motility assay, and invasion assay), whereas the other cell lines were either completely non-responsive (Hs746T and LMSU cells, non-responders) or sensitive in certain assays (AGS cells, partial responder). We conclude that the definition of response or non-response to treatment strongly depended on the phenotypic assay (Table 1).

### Phenotypic characterization of EGFR signaling effects on cellular motility, morphology and invasive capacity

In the present study, we concentrated on the cellular motility analysis and determined the surrogate markers “percentage of motile cells” and “average speed”. The 2D motility of tumor cells on flat dishes in the absence of an attractant concentration gradient has been described as a random walk [11], whereas three-dimensional (3D) migration through a complex environment did not follow a random walk [34]. Among the key descriptors of the 2D migration behaviors were the speed of the cells and the directional persistence, properties that were influenced by environmental stimuli such as growth factors [11]. Targeting tumor cell motility, which is a prerequisite for the fatal dissemination of tumor cells in the body, has been shown as a strategy against invasion and metastasis [11].

We showed that EGF-induced increases in the percentage of motile cells or average speed were reverted by concomitant cetuximab treatment in MKN1 or in AGS cells, respectively. Therefore, these cell lines were defined as cetuximab-responsive with regard to their motile behavior at least for one descriptor. In contrast, Hs746T and LMSU cells were not sensitive to cetuximab treatment in the motility assay. We used a semi-automatic, time-extensive method for the determination of the descriptors of cellular motility in a total of 1500 cells. One might argue that the use of fully automatic cell tracking software [35] would be preferable. However, cancer cells in phase-contrast microscopy images have complex appearances with irregular shapes. In addition, the cells often aggregate into clusters, which are difficult to separate by a detection or tracking algorithm. Although detectors may be able to address the latter problem on its own [36], the combination of both problems is still challenging. Therefore, software for fully automated cell detection and tracking was not available at the time of the analysis. Nevertheless, we previously showed that the semi-automatic method that we used as a substitute, produced reliable results when we analyzed the effect of inhibitors or siRNA against EGFR [14, 37], MMP3 [38] or the Rho GTPases Rac1 and Rho [39] in cancer cells. However, our work on the automatic detection of cell nuclei and cell tracking is ongoing [40].

Morphologically, lamellipodia and few filopodia were observed in motile, untreated MKN1 cells. Addition of EGF led to enhanced lamellipodia and filopodia formation. In MKN1 cells treated with EGF and additional cetuximab, the protrusions were reduced to a level similar to untreated cells. MKN1 cells treated with cetuximab-alone therapy exhibited fewer filopodia compared to untreated MKN1 cells. In Hs746T cells, no morphological differences were detectable upon different treatments. These results are consistent with the analyses by Felkl et al. [41], showing the EGF-induced temporal and spatial coordination of cytoskeletal filament reorganization and focal ECM adhesion dynamics. An increase in lamellipodial activity was detected within minutes of EGF treatment, persisted for up to 1 h and was inhibited by cetuximab application. Hs746T cells did not respond to either EGF or cetuximab treatment.

A significant increase in the number of invasive MKN1 cells was observed following EGF treatment and was reduced by concomitant application of cetuximab in a concentration-dependent manner. In contrast, no significant changes were observed in AGS, Hs746T and LMSU cells. Interestingly, the invasive capacity of MKN1, in contrast to Hs746T cells (Fig. 3), was reflected by lamellipodia formation, as shown by Felkl et al. [41]. Thus, lamellipodia formation is an essential step for cell migration, which is required for tumor cell invasiveness. Our findings that MKN1 invasion is EGF-inducible are supported by another study [42]. The inhibition of EGF-induced invasion and ruffling from cetuximab treatment further highlighted that MKN1 cells responded to cetuximab treatment not only on the molecular but also on the phenotypic level.

### Time-resolved activation of EGFR, MAPK and AKT

We hypothesized that proteins that are functionally involved in the mediation of phenotypic features can be identified by their reaction to EGF and/or cetuximab treatment. Therefore, we analyzed the activation profiles of EGFR and the downstream kinases MAPK and AKT to link the motile and invasive behavior of the cells with molecular characteristics. Investigation of the activation status of EGFR, MAPK and AKT by Western blot analysis showed transient activation of EGFR by EGF treatment in the cetuximab-sensitive cell line MKN1, which was reverted by concomitant cetuximab treatment. MAPK activation and minor AKT activation were induced by EGF treatment in MKN1 cells; however, this induction was not fully reversed by additional cetuximab treatment under the chosen experimental conditions. We speculated that the strong reduction in the activation of the EGFR after 4 h cetuximab treatment that was partially transmitted to the downstream kinase MAPK led to the strong reduction of the number of motile cells in MKN1 cells.

In contrast, the activity of AKT and MAPK was not influenced in the cell line Hs746T, which was chosen as an example of a cetuximab non-responsive cell line. One possible explanation for the observation that cetuximab had no significant effect on the activation of EGFR, MAPK or AKT in Hs746T cells could be that the *MET* amplification of this cell line [33] leads to ERBB3 phosphorylation and PI3K activation in an EGFR- and ERBB2-independent manner by a mechanism described by Engelmann et al. [43].

### Analysis of EGFR downstream signaling by proteome profiler analysis

**Fig. 8:**
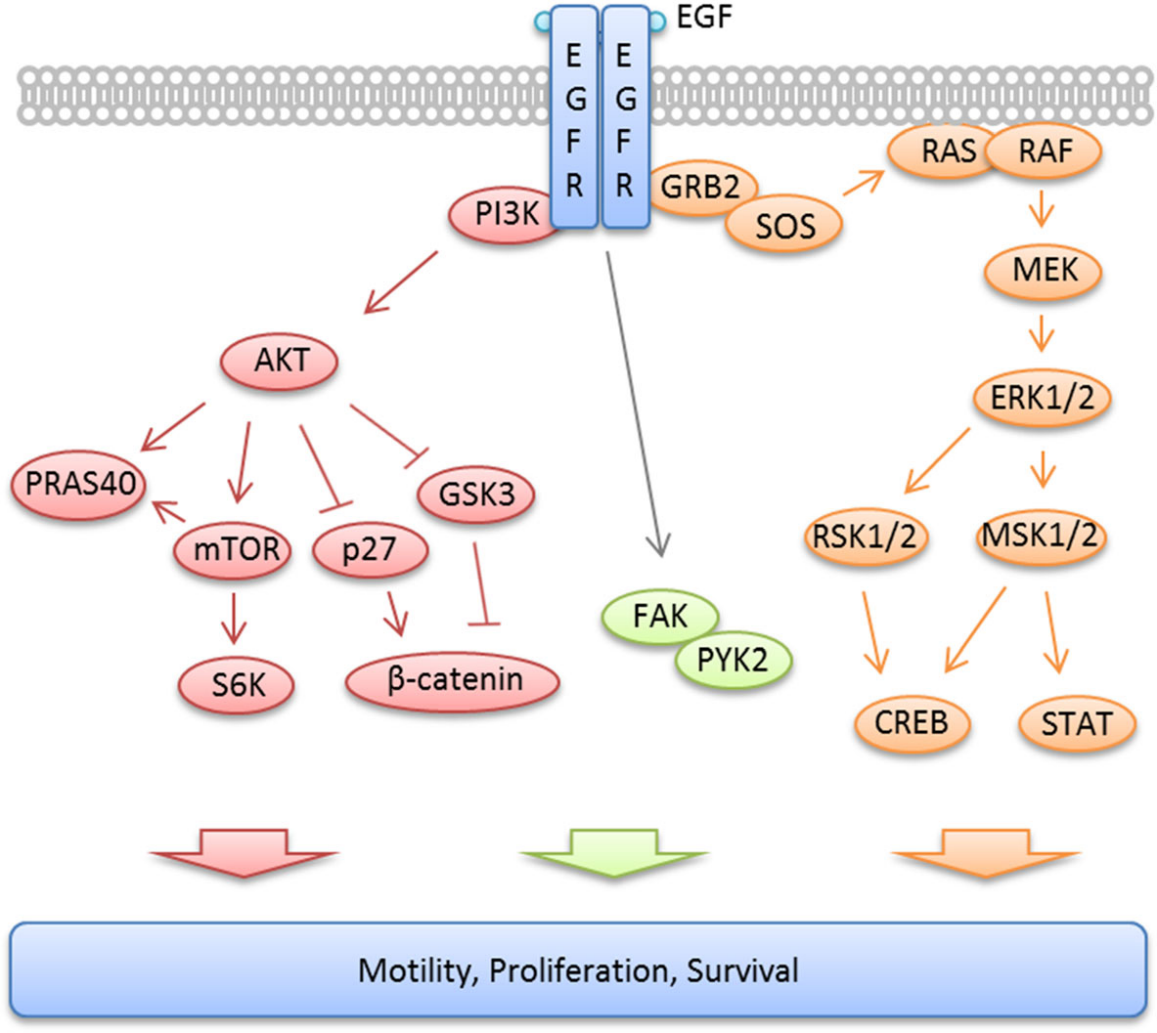
Schematic EGFR signaling pathway. EGFR and the main downstream signaling molecules that were analyzed in this study by proteome profiler and/or Western blot analyses are shown

Next, we further analyzed kinases downstream of EGFR signaling. Therefore, we used a proteome profiler assay to screen for branches of the EGFR signaling pathway that were highly affected by cetuximab treatment. To validate the results, we performed Western blot analysis for four phospho-proteins: pAKT, pCREB, pFAK and pMAPK. As shown in Fig. 8 and Additional file 4: Figure S3, the analyzed proteins are involved in the network of EGFR signaling that results in the regulation of cellular motility.

MKN1 cells showed an EGF/cetuximab-responsive profile for several components of the MAPK signaling branch (pMAPK, pMSK1/2, pRSK1/2/3, pCREB) as well as the AKT signaling pathway (pAKT, pTOR) in the proteome profiler analysis. This observation suggests that the EGFR activation and inhibition by cetuximab is transmitted to both branches of the pathway. In contrast, no such trend of EGF/cetuximab-responsive profiles was detected for the non-responsive cell line Hs746T. In the validation Western blot analysis of MKN1 cells, pAKT and pMAPK showed a clear EGF/cetuximab-responsive phosphorylation profile. This was not observed for the cetuximab-resistant cell line Hs746T. In comparison to the time-resolved activation profiling, the treatment effects were far more prominent in the proteome profiler, especially for pAKT. One likely explanation for these findings is that we used serum-starvation conditions for the proteome profiler experiments. Therefore, these different findings were expected.

An EGF/cetuximab-responsive phosphorylation profile for the transcription factor CREB was observed in MKN1 cells but not in cetuximab-resistant Hs746T cells. CREB is activated via MAPK in gastric cancer cells upon gastrin stimulation [44]. Our results indicate that EGF stimulation has a similar effect on CREB activity in gastric cancer cell lines. Interestingly, in contrast to MKN1 cells, AGS cells displayed an EGF/cetuximab-responsive phosphorylation profile for pMAPK but not pCREB. We believe that certain phenotypic differences observed between MKN1 and AGS cells were due to this important difference. CREB is an important transcription factor that can bind to the promoter regions of several thousand genes [45, 46]. However, although CREB activation was strongly inhibited after cetuximab treatment in MKN1 cells, the effect of cetuximab in the phenotypic assays was relatively moderate. Interestingly, the tumor suppressor protein p53 was strongly activated in MKN1 cells but not in Hs746T cells. EGF and cetuximab did not influence the activation of p53.

FAK is a regulator of cell migration (for review [25]), and increased FAK activation has been linked with enhanced invasiveness in gastric cancer [47]. Therefore, this phospho-protein was of special interest for our study. Our experiments revealed that, although FAK phosphorylation increased upon EGF application, FAK activation was not responsive to cetuximab treatment. These results could partially explain the moderate effect of cetuximab on cell motility in MKN1 cells.

### Involvement of genetic alterations in the cetuximab response

As mentioned before, genetic alterations in the EGFR signaling network as well as interaction partners of EGFR play important roles in the response to EGFR-targeted therapies [5–7]. We previously demonstrated that activation of MET and mutations in *KRAS* or *CDH1* were linked to the cetuximab insensitivity in gastric cancer cell lines [6, 7].

For two of the gastric cancer cell lines analyzed in this study, genetic defects concerning *KRAS* have been published. These previous reports included the MKN1 cell line, which exhibited *KRAS* amplification, and the AGS cell line, which expressed an activating G12D mutation in *KRAS* [48, 49]. In contrast to colorectal cancers, activating mutations in *KRAS* are a rare event in gastric cancer, present in approximately 5% of tumors [50]. With a frequency of approximately 9%, *KRAS* amplifications are slightly more common [3]. This is of special interest because *KRAS* amplifications in colorectal cancers were shown to be associated with resistance to EGFR inhibitors, including cetuximab [51]. However, there was no association with response for MKN1, as this cell line is cetuximab-sensitive.

As recently described by our group, AGS is cetuximab-resistant for cell proliferation [6]. In the time-lapse microscopy studies presented here, the average speed of AGS cells increased after EGF application, and this increase was inhibited by concomitant cetuximab treatment. This indicates that the EGFR signaling branch affecting cell migration was less influenced by KRAS than cell proliferation. Taken together, our studies indicate that *KRAS* amplifications and *KRAS* mutations are of less importance for predicting cetuximab sensitivity in gastric cancer, especially regarding cell migration.

## Conclusion

Our results suggest that different components of the EGFR signaling network are important regulators of the phenotypic and molecular behavior of gastric cancer cell lines in response to cetuximab treatment. We showed that the definition of response or non-response to treatment strongly depended on the type of phenotypic assay. Significant reversion of the EGF-induced effect on the phenotypic and molecular level was observed in the responder cell lines, whereas non-responder cell lines did not significantly react to either EGF or cetuximab. This suggests that, in the non-responder cell lines, the EGFR pathway is either nonfunctional or fully activated and not susceptible.

## Additional files

Additional file 1: Tables S1–S7. Additional statistical data. Comprehensive statistical data, including mean and *p*-values from statistical analysis. (PDF 252 kb)

Additional file 2: Figure S1. Morphological differences in MKN1 cells after treatment with EGF and/or cetuximab. Denoted are hourly time-lapse microscopy movies of MKN1 cells with different treatments: untreated, EGF (5 ng/ml), EGF (5 ng/ml) + cetuximab (1 μg/ml) and cetuximab (1 μg/ml). Additionally, images of Hs746T cells after 4 h treatment are shown. The arrowheads indicate filopodia (1) and lamellipodia formation (2). Scale bar = 50 μm. (PDF 366 kb)

Additional file 3: Figure S2. Arrangement of the kinases in the kinase proteome profiler assay, indicating the specific phosphorylation sites. (PDF 206 kb)

Additional file 4: Figure S3. Network connecting EGFR signaling to the regulation of cellular motility. A total of 179 nodes from KEGG, Reactome, BioModels and the literature are implicated in EGFR signaling or cellular motility regulation events. Each node represents one to many functionally equivalent proteins (e.g., node “PIK3R5” represents the 12 proteins PIK3R5, PIK3C2A, PIK3C2B, PIK3C2G, PIK3C3, PIK3CA, PIK3CB, PIK3CD, PIK3CG, PIK3R1, PIK3R2, PIK3R3). The potential flow of signals is indicated by protein modification, protein-protein interaction and transcriptional regulation events extracted from KEGG, Reactome, DIP, IntAct, MiMI and ITFP. A subset of proteins and their associations were manually enlarged to emphasize either involvement in standard EGFR signaling and motility regulation pathways or because they are required to connect standard components. Thus far, the data available in the standard pathway resources did not provide a direct mechanistic explanation as to how EGFR signaling might influence cellular motility. While such a mechanism can be proposed based on standard components and added information about PPI, there are potentially many alternative flows through the network that can provide alternative or preferred routes. Green arrows represent phosphorylation and direction of activation. Red barred lines represent direction of inhibition. Black, single-arrowhead arrows represent associations with unspecified functional directions. Double-headed arrows represent undirected protein-protein interactions. (PDF 674 kb)

## Abbreviations

Cet: Cetuximab
DAPI: 4′,6-diamidino-2-phenylindole
DMEM: Dulbecco’s Modified Eagle Medium
EGF: Epidermal growth factor
EGFR: Epidermal growth factor receptor
FBS: Fetal bovine serum
HER2: Human epidermal growth factor receptor 2
IU: International units
MAPK: Mitogen-activated protein kinase
MEM: Minimum Essential Medium Eagle
pAKT: Phosphorylated AKT
PBS: Phosphate-buffered saline
pEGFR: Phosphorylated epidermal growth factor receptor
PI3K: Phosphatidylinositol 3-kinase
pMAPK: Phosphorylated mitogen-activated protein kinase
PPI: Protein-protein interaction
RTK: Receptor tyrosine kinase
SD: Standard deviation
WB: Western blot
