# Supplementary material for: Evaluation of epidermal growth factor receptor signaling effects in gastric cancer cell lines by detailed motility-focused phenotypic characterization linked with molecular analysis
Source: BMC Cancer. 2017 Dec 13;17:845. doi: 10.1186/s12885-017-3822-3 (PMC5729506; doi:10.1186/s12885-017-3822-3)
Supplement: Supplementary file 1 — Additional statistical data. Comprehensive statistical data, including mean and p-values from statistical analysis. (PDF 252 kb) [file 12885_2017_3822_MOESM1_ESM.pdf]

Table S1

Analysis of the 2D motility parameters “percentage of motile cells” and “average speed” for AGS, Hs746T, LMSU and MKN1 cells. The numbers of analyzed films (n), the mean and the standard deviation (SD) of the percentage of motile cells (motility) and the average speed are shown

**AGS**	**Untreated**	**EGF [5 ng/ml]**	**EGF + Cetuximab [1 µg/ml]**	**EGF + Cetuximab [50 µg/ml]**	**Cetuximab [1 µg/ml]**
**n**	5	4	4	5	3
**Motility [%]**					
Mean	96.26	99.43	96.87	96.78	96.44
SD	3.82	1.14	3.19	3.81	1.94
**Speed [µm/h]**					
Mean	45.00	61.33	48.91	48.94	41.02
SD	9.82	7.03	4.00	12.42	7.76

**Hs746T**	**Untreated**	**EGF [5 ng/ml]**	**EGF + Cetuximab [1 µg/ml]**	**EGF + Cetuximab [50 µg/ml]**	**Cetuximab [1 µg/ml]**
**n**	8	8	8	7	5
**Motility [%]**					
Mean	86.84	84.16	83.19	86.25	79.64
SD	8.85	11.02	8.56	9.71	12.57
**Speed [µm/h]**					
Mean	39.73	38.69	36.90	41.96	43.19
SD	8.30	11.06	7.54	7.05	5.63

**LMSU**	**Untreated**	**EGF [5 ng/ml]**	**EGF + Cetuximab [1 µg/ml]**	**EGF + Cetuximab [50 µg/ml]**	**Cetuximab [1 µg/ml]**
**n**	3	3	3	3	3
**Motility [%]**					
Mean	68.29	79.52	63.55	69.49	67.88
SD	2.40	5.98	36.28	16.22	10.56
**Speed [µm/h]**					
Mean	35.44	38.99	36.36	40.48	37.97
SD	4.34	3.96	7.34	7.16	0.75

**MKN1**	**Untreated**	**EGF [5 ng/ml]**	**EGF + Cetuximab [0.05 µg/ml]**	**EGF + Cetuximab [0.1 µg/ml]**	**EGF + Cetuximab [1 µg/ml]**	**Cetuximab [1 µg/ml]**
**n**	5	7	6	6	6	5
**Motility [%]**						
Mean	49.81	54.25	43.71	43.06	38.23	32.48
SD	5.04	8.70	8.52	8.19	10.13	15.46
**Speed [µm/h]**						
Mean	13.88	17.21	15.61	15.60	14.43	13.98
SD	1.62	3.10	1.76	3.14	3.61	3.06

Table S2

Pairwise comparisons of the parameter "percentage of motile cells" between different treatment conditions for AGS, Hs746T, LMSU and MKN1 cells. Cells were treated with EGF and/or cetuximab. Welch t-test was used. Significant p-values are shown.

Abbreviations: EGF 5: 5 ng/ml EGF; Cet 0.1: 0.1 µg/ml cetuximab; Cet 1: 1 µg/ml cetuximab; Cet 50: 50 µg/ml cetuximab

Cell line	Groups (X vs. Y)<br>X	 Y	Mean ± SD (X)	Mean ± SD (Y)	p-value
**MKN1**	-	EGF 5 + Cet 1	49.81 ± 5.04	38.23 ± 10.13	0.041
	EGF 5	EGF 5 + Cet 0.1	54.25 ± 8.70	43.06 ± 8.19	0.036
	EGF 5	EGF 5 + Cet 1	54.25 ± 8.70	38.23 ± 10.13	0.013
	EGF 5	Cet 1	54.25 ± 8.70	32.48 ± 15.46	0.030

Table S3

Pairwise comparisons of the parameter "average speed" between different treatment conditions for AGS, Hs746T, LMSU and MKN1 cells. Cells were treated with EGF and/or cetuximab. Welch t-test was used. Significant p-values are shown.

Abbreviations: EGF 5: 5 ng/ml EGF; Cet 1: 1 µg/ml cetuximab

Cell line	Groups (X vs. Y)<br>X	 Y	Mean ± SD (X)	Mean ± SD (Y)	p-value
**AGS**	-	EGF 5	45.00 ± 9.82	61.33 ± 7.03	0.023
	EGF 5	EGF 5 + Cet 1	61.33 ± 7.03	48.91 ± 4.00	0.030
	EGF 5	Cet 1	61.33 ± 7.03	41.02 ± 7.76	0.022
**MKN1**	-	EGF 5	13.88 ± 1.62	17.21 ± 3.10	0.038

Table S4

Pairwise comparisons of the invasion potential between different treatment conditions for AGS, Hs746T, LMSU and MKN1 cells. Cells were treated with EGF and/or cetuximab. Two-sided Welch t-test and one-sample t-test were used. Significant p-values are shown.

Abbreviations: EGF 5: 5 ng/ml EGF; Cet 0.1: 0.1 µg/ml cetuximab; Cet 1: 1 µg/ml cetuximab; Cet 50: 50 µg/ml cetuximab

Cell line	Groups (X vs. Y) X	Y	Mean ± SD (X)	Mean ± SD (Y)	p-value
**AGS**	-	Cet 1	100.000	141.939 ± 13.258	0.032
	EGF 5	Cet 1	89.362 ± 15.654	141.939 ± 13.258	0.012
**MKN1**	-	EGF 5	100.000	522.327 ± 177.368	0.006
	-	EGF 5 + Cet 0.1	100.000	349.438 ± 188.038	0.041
	EGF 5	EGF 5 + Cet 1	522.327 ± 177.368	159.823 ± 81.449	0.007
	EGF 5	Cet 1	522.327 ± 177.368	88.069 ± 19.558	0.005
	EGF 5 + Cet 0.1	Cet 1	349.438 ± 188.038	88.069 ± 19.558	0.035

Table S5

Pairwise comparisons of the EGFR, MAPK and AKT activation between different treatments in MKN1 cells. Cells were treated with EGF and/or cetuximab. Welch t-test was used. Significant p-values of relevant comparisons are shown.

Abbreviations: EGF 5: 5 ng/ml EGF; Cet 0.05: 0.05 µg/ml cetuximab; Cet 0.1: 0.1 µg/ml cetuximab; Cet 1: 1 µg/ml cetuximab; Cet 10: 10 µg/ml cetuximab

Activation of protein		Groups (X vs. Y) X	Y	Mean ± SD (X)	Mean ± SD (Y)	p-value
**EGFR**	1 min	EGF 5 + Cet 0.05	EGF 5 + Cet 10	1.991 ± 0.196	0.669 ± 0.326	0.007
			Cet 0.05	1.991 ± 0.196	0.263 ± 0.056	0.002
		EGF 5 + Cet 0.1	EGF 5 + Cet 10	2.219 ± 0.345	0.669 ± 0.326	0.005
			Cet 0.1	2.219 ± 0.345	0.236 ± 0.053	0.009
	3 min	EGF 5	EGF 5 + Cet 1	2.478 ±0.449	1.056 ± 0.317	0.014
			EGF 5 + Cet 10	2.478 ±0.449	0.472 ± 0.126	0.012
			Cet 10	2.478 ±0.449	0.118 ± 0.066	0.011
		EGF 5 + Cet 0.05	EGF 5 + Cet 1	3.137 ± 0.763	1.056 ± 0.317	0.028
			EGF 5 + Cet 10	3.137 ± 0.763	0.472 ± 0.126	0.024
			Cet 0.05	3.137 ± 0.763	0.175 ± 0.0418	0.021
		EGF 5 + Cet 0.1	EGF 5 + Cet 10	1.991 ± 0.530	0.472 ± 0.126	0.032
			Cet 0.1	1.991 ± 0.530	0.149 ± 0.050	0.026
		EGF 5 + Cet 1	Cet 0.1	1.056 ± 0.317	0.149 ± 0.050	0.036
		EGF 5 + Cet 10	Cet 10	0.472 ± 0.126	0.118 ± 0.066	0.023
	15 min	EGF 5	EGF 5 + Cet 1	2.808 ± 0.250	0.860 ± 0.492	0.009
			EGF 5 + Cet 10	2.808 ± 0.250	0.376 ± 0.076	0.002
		EGF 5 + Cet 0.05	EGF 5 + Cet 1	2.293 ± 0.407	0.860 ± 0.492	0.019
			EGF 5 + Cet 10	2.293 ± 0.407	0.376 ± 0.076	0.012

			Cet 0.05	2.293 ± 0.407	0.154 ± 0.025	0.012
		EGF 5 + Cet 0.1	EGF 5 + Cet 10	1.939 ± 0.607	0.376 ± 0.076	0.045
			Cet 0.1	1.939 ± 0.607	0.128 ± 0.049	0.035
		EGF 5 + Cet 10	Cet 10	0.376 ± 0.076	0.163 ± 0.095	0.041
	4 h	EGF 5 + Cet 0.05	Cet 0.05	2.249 ± 0.801	0.311 ± 0.167	0.047
**MAPK**	3 min	EGF 5 + Cet 10	Cet 10	1.113 ± 0.166	0.619 ± 0.184	0.026
	15 min	EGF 5 + Cet 0.05	Cet 0.05	1.549 ± 0.377	0.727 ± 0.316	0.046
		EGF 5 + Cet 0.1	Cet 0.1	1.544 ± 0.215	0.491 ± 0.089	0.007
		EGF 5 + Cet 1	Cet 1	1.503 ± 0.448	0.315 ± 0.207	0.028
		EGF 5 + Cet 10	Cet 10	1.263 ± 0.186	0.372 ± 0.389	0.040
	4 h	EGF 5 + Cet 0.1	Cet 0.1	1.467 ± 0.435	0.274 ± 0.205	0.026
		EGF 5 + Cet 1	Cet 1	1.438 ± 0.257	0.223 ± 0.137	0.005
**AKT**	1 min	0	Cet 10	0.881 ± 0.014	1.056 ± 0.067	0.040
	3 min	EGF 5 + Cet 0.05	Cet 0.05	1.361 ± 0.189	0.779 ± 0.088	0.019
	15 min	EGF 5 + Cet 0.05	Cet 0.05	1.578 ± 0.453	0.549 ± 0.335	0.038
		EGF 5 + Cet 0.1	EGF 5 + Cet 10	1.379 ± 0.185	0.913 ± 0.151	0.029
			Cet 0.1	1.379 ± 0.185	0.502 ± 0.152	0.004
		EGF 5 + Cet 1	Cet 1	1.333 ± 0.333	0.516 ± 0.147	0.035

Table S6

Pairwise comparisons of the EGFR, MAPK and AKT activation between different treatments in Hs746T cells. Cells were treated with EGF and/or cetuximab. Welch t-test was used. Significant p-values of relevant comparisons are shown.

Abbreviations: EGF 5: 5 ng/ml EGF; Cet 1: 1 µg/ml cetuximab; Cet 50: 50 µg/ml cetuximab

Activation of protein		Groups (X vs. Y) X	Y	Mean ± SD (X)	Mean ± SD (Y)	p-value
**AKT**	3 min	EGF 5	EGF 5 + Cet 1	1.169 ± 0.050	0.950 ± 0.088	0.030
			EGF 5 + Cet 50	1.169 ± 0.050	0.971 ± 0.026	0.008
	4 h	0	EGF 5	0.812 ± 0.059	1.113 ± 0.038	0.003
			Cet 1	0.812 ± 0.059	0.961 ± 0.051	0.031

Table S7

Statistical values of activation of the EGFR signaling components pAKT, pCREB, pFAK and pMAPK in the gastric cancer cell lines Hs746T and MKN1.

Cells were treated for 4h with EGF and/or cetuximab. Listed are all relevant and significant values. The Welch t-test was used to compare values.

Abbreviations: EGF 30: 30 ng/ml EGF; Cet 1: 1 µg/ml cetuximab

**Activation of protein**	**Cell line**	**Groups (X vs. Y)** X	Y	**Mean ± SD (X)**	**Mean ± SD (Y)**	**p-value**
**AKT**	Hs746T	EGF 30 + Cet 1	Cet 1	0.881 ± 0.173	1.207 ± 0.136	0.043
	MKN1	-	EGF 30	0.973 ± 0.173	1.569 ± 0.068	0.017
		EGF 30	EGF 30 + Cet 1	1.569 ± 0.068	0.997 ± 0.075	0.001
**CREB**	Hs746T	-	EGF 30	1.225 ± 0.205	0.726 ± 0.092	0.036
		-	EGF 30 + Cet 1	1.225 ± 0.205	0.756 ± 0.198	0.046
	MKN1	-	Cet1	1.077 ± 0.188	0.453 ± 0.075	0.018
**FAK**	MKN1	-	EGF 30	0.844 ± 0.175	1.218 ± 0.104	0.044
		-	EGF 30 + Cet 1	0.844 ± 0.175	1.257 ± 0.108	0.034
**MAPK**	Hs746T	EGF 30	EGF 30 + Cet 1	1.131 ± 0.095	0.882 ± 0.041	0.030
	MKN1	-	EGF 30	0.938 ± 0.125	1.475 ± 0.120	0.006
		-	Cet 1	0.938 ± 0.125	0.238 ± 0.206	0.012
