# Supplementary material for: Evaluation of epidermal growth factor receptor signaling effects in gastric cancer cell lines by detailed motility-focused phenotypic characterization linked with molecular analysis
Source: BMC Cancer. 2017 Dec 13;17:845. doi: 10.1186/s12885-017-3822-3 (PMC5729506; doi:10.1186/s12885-017-3822-3)
Supplement: Supplementary file 2 — Morphological differences in MKN1 cells after treatment with EGF and/or cetuximab. Denoted are hourly time-lapse microscopy movies of MKN1 cells with different treatments: untreated, EGF (5 ng/ml), EGF (5 ng/ml) + cetuximab (1 μg/ml) and cetuximab (1 μg/ml). Additionally, images of Hs746T cells after 4 h treatment are shown. The arrowheads indicate filopodia (1) and lamellipodia formation (2). Scale bar = 50 μm. (PDF 366 kb) [file 12885_2017_3822_MOESM2_ESM.pdf]

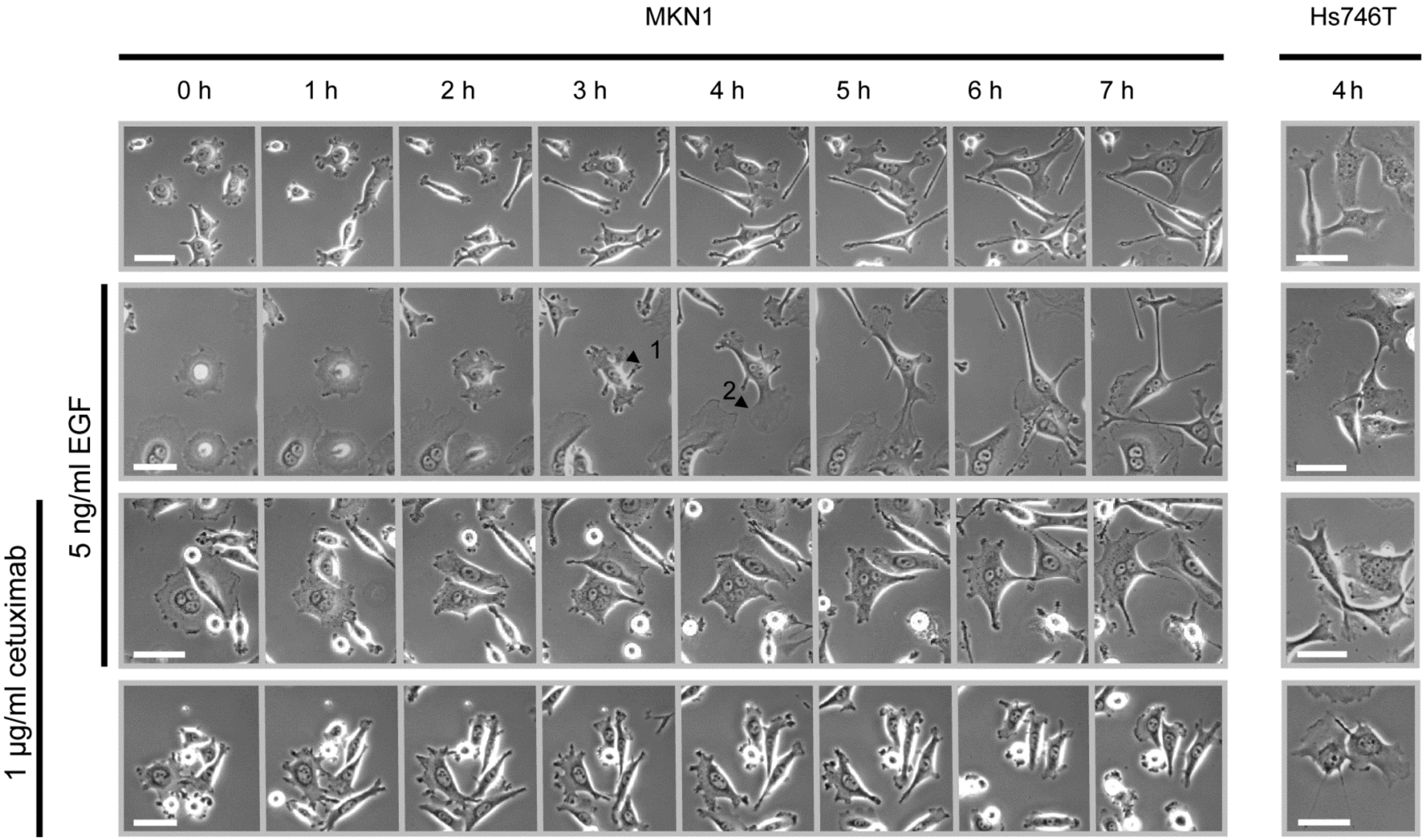

Figure S1: Morphological differences in MKN1 cells after treatment with EGF and/or cetuximab during a 7 h film. Denoted are hourly time-lapse microscopy film pictures of MKN1 cells with different treatments: untreated, EGF (5 ng/ml), EGF (5 ng/ml) + cetuximab (1 µg/ml) and cetuximab (1 µg/ml). Additionally, pictures of Hs746T cells after 4 h treatment are shown. The arrowheads indicate filopodia (1) and lamellipodia formation (2). Scale bar = 50 µm.
