# Supplementary material for: Evaluation of epidermal growth factor receptor signaling effects in gastric cancer cell lines by detailed motility-focused phenotypic characterization linked with molecular analysis
Source: BMC Cancer. 2017 Dec 13;17:845. doi: 10.1186/s12885-017-3822-3 (PMC5729506; doi:10.1186/s12885-017-3822-3)
Supplement: Supplementary file 3 — Arrangement of the kinases in the kinase proteome profiler assay, indicating the specific phosphorylation sites. (PDF 206 kb) [file 12885_2017_3822_MOESM3_ESM.pdf]

Figure S2

	1	2	3	4	5	6	7	8	9	10	11	12	13	14	15	16	17	18
A	+		**p38α**<br>T180/Y182		**ERK1/2**<br>T202/Y204,<br>T185/Y187		**JNK pan**<br>T183/Y185,<br>T221/Y223		**GSK-3α/β**<br>S21/S9				**p53**<br>S392				+	
B			**MEK1/2**<br>S218/S222<br>S222/S226		**MSK1/2**<br>S376/S360		**AMPKα1**<br>T174		**Akt1/2/3**<br>S473		**Akt1/2/3**<br>T308		**p53**<br>S46					
C	**TOR**<br>S2448		**CREB**<br>S133		**HSP27**<br>S78/S82		**AMPKα2**<br>T172		**β-Catenin**<br>-		**p70 S6 Kinase**<br>T389		**p53**<br>S15		**p27**<br>T198		**Paxillin**<br>Y118	
D	**Src**<br>Y419		**Lyn**<br>Y397		**Lck**<br>Y394		**STAT2**<br>Y689		**STAT5a**<br>Y699		**p70 S6 Kinase**<br>T421/S424		**RSK1/2/3**<br>S380		**p27**<br>T157		**PLCγ-1**<br>Y783	
E	**Fyn**<br>Y420		**Yes**<br>Y426		**Fgr**<br>Y412		**STAT3**<br>Y705		**STAT5b**<br>Y699		**p70 S6 Kinase**<br>T229		**RSK1/2**<br>S221		**C-Jun**<br>S63		**Pyk2**<br>Y402	
F	**Hck**<br>Y411		**Chk-2**<br>T68		**FAK**<br>Y397		**STAT6**<br>Y641		**STAT 5a/b**<br>Y699		**STAT1**<br>Y701		**STAT4**<br>Y693		**eNOS**<br>s1177		**PBS**	
G	+				**PBS**													
