# Supplementary material for: Evaluation of epidermal growth factor receptor signaling effects in gastric cancer cell lines by detailed motility-focused phenotypic characterization linked with molecular analysis
Source: BMC Cancer. 2017 Dec 13;17:845. doi: 10.1186/s12885-017-3822-3 (PMC5729506; doi:10.1186/s12885-017-3822-3)
Supplement: Supplementary file 4 — Network connecting EGFR signaling to the regulation of cellular motility. A total of 179 nodes from KEGG, Reactome, BioModels and the literature are implicated in EGFR signaling or cellular motility regulation events. Each node represents one to many functionally equivalent proteins (e.g., node “PIK3R5” represents the 12 proteins PIK3R5, PIK3C2A, PIK3C2B, PIK3C2G, PIK3C3, PIK3CA, PIK3CB, PIK3CD, PIK3CG, PIK3R1, PIK3R2, PIK3R3). The potential flow of signals is indicated by protein modification, protein-protein interaction and transcriptional regulation events extracted from KEGG, Reactome, DIP, IntAct, MiMI and ITFP. A subset of proteins and their associations were manually enlarged to emphasize either involvement in standard EGFR signaling and motility regulation pathways or because they are required to connect standard components. Thus far, the data available in the standard pathway resources did not provide a direct mechanistic explanation as to how EGFR signaling might influence cellular motility. While such a mechanism can be proposed based on standard components and added information about PPI, there are potentially many alternative flows through the network that can provide alternative or preferred routes. Green arrows represent phosphorylation and direction of activation. Red barred lines represent direction of inhibition. Black, single-arrowhead arrows represent associations with unspecified functional directions. Double-headed arrows represent undirected protein-protein interactions. (PDF 674 kb) [file 12885_2017_3822_MOESM4_ESM.pdf]

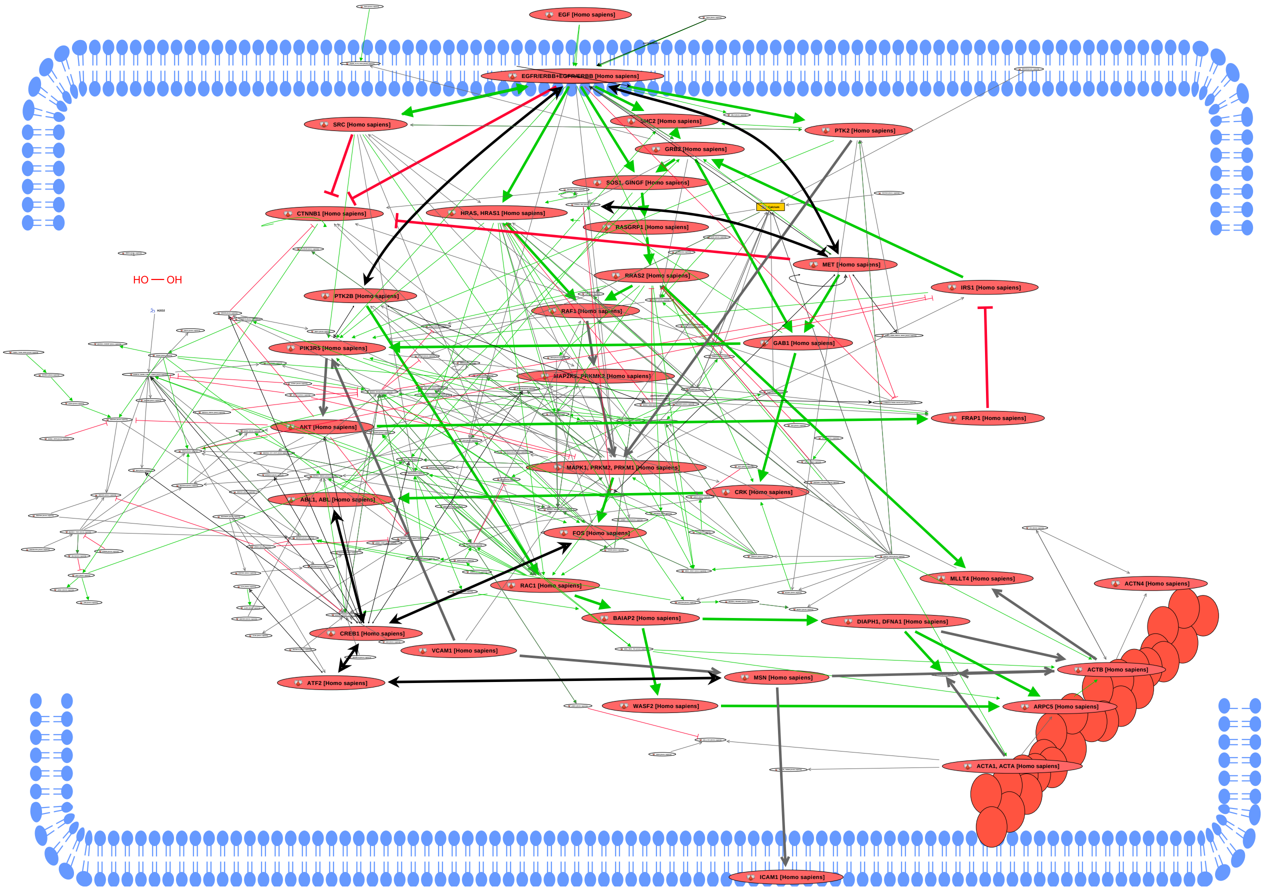

Figure S3: Network connecting EGFR signaling to the regulation of cellular motility. A total of 179 nodes from KEGG, Reactome, BioModels and the literature are implicated in EGFR signaling or cellular motility regulation events. Each node represents one to many functionally equivalent proteins (e.g., node "PIK3R5" represents the 12 proteins PIK3R5, PIK3C2A, PIK3C2B, PIK3C2G, PIK3C3, PIK3CA, PIK3CB, PIK3CD, PIK3CG, PIK3R1, PIK3R2, PIK3R3. The potential flow of signals is indicated by protein modification, protein-protein interaction and transcriptional regulation events extracted from KEGG, Reactome, DIP, IntAct, MiMI and ITFP. A subset of proteins and their associations were manually enlarged to emphasize either involvement in standard EGFR signaling and motility regulation pathways or because they are required to connect standard components. Thus far, information available in the standard pathway resources did not provide a direct mechanistic explanation as to how EGFR signaling might influence cellular motility. While such a mechanism can be proposed based on standard components and added information about PPI, there are potentially many alternative flows through the network that can provide alternative or preferred routes. Green arrows represent phosphorylation and direction of activation. Red barred lines represent direction of inhibition. Black, single-arrowhead arrows represent associations with unspecified functional directions. Double-headed arrows represent undirected protein-protein interactions.
